# Association Between Advanced Age and Perioperative Complications Among Women Operated for Pelvic Organ Prolapse in Urogynecology Camps in Uganda

**DOI:** 10.7759/cureus.108822

**Published:** 2026-05-13

**Authors:** Kalyebara Paul Kato, Joseph Ngonzi, Onesmus Byamukama, Brenda Ainomugisha, Wilson Birungi, Rogers Kajabwangu, Verena Geissbuehler, Musa Kayondo

**Affiliations:** 1 Obstetrics and Gynecology, Mbarara University of Science and Technology, Mbarara, UGA; 2 Obstetrics and Gynaecology, Mbarara Regional Referral Hospital, Mbarara, UGA; 3 Obstetrics and Gynaecology, University of Basel, Basel, CHE

**Keywords:** complications, elderly women, pelvic organ prolapse, surgery, uganda

## Abstract

Background: The burden of pelvic organ prolapse (POP) is the highest among elderly women compared to their young and middle-aged counterparts. These women are also faced with various medical comorbidities. Surgery in these women presents a challenge due to the increased risk of perioperative complications, especially in low-resource settings. Therefore, this study aimed to determine the association between advanced age and perioperative complications among women undergoing POP surgery in a resource-limited setting.

Methods: This was a retrospective review of records for women who had surgery for POP during urogynecology camps conducted over a four-year period from January 2022 to December 2025 at eight centers in Uganda. A woman was considered elderly if she was aged 70 years and above. Perioperative complications assessed included intraoperative injury to pelvic viscera, duration of hospital stay, and in-hospital adverse events. We compared the proportions of perioperative complications in the elderly and non-elderly using a chi-square test. Cramér's V coefficient was used to assess effect size. Bivariate and multivariate modified Poisson regression analyses were done to determine the association between advanced age and perioperative complications. The level of statistical significance was set at <0.05.

Results: A total of 547 women underwent surgery for POP during the study period, of which 63 (11.5%) were elderly and 484 (88.5%) were non-elderly. Elderly women had a higher proportion of hypertension (26/63, 41.3% vs. 111/484, 22.9%), stage IV POP (29/63, 46% vs. 110/484, 22.7%), and POP surgery in two compartments (46/63, 73% vs. 184/484, 38.1%) (p < 0.005). The overall prevalence of perioperative complications was 14.26% (n = 78, CI: 11.57-17.46). There was no significant difference in the proportion of perioperative complications between the two groups (10/63, 15.9% vs. 68/484, 14.1%) (p = 0.697). At the multivariate analysis, advanced age was not associated with perioperative complications (aPR 1.07; CI: 0.57-2.03; p = 0.828).

Conclusions: Advanced age was not associated with perioperative complications. We recommend that surgery be offered as a viable option to elderly women with POP in urogynecological surgical camps and enhanced recovery after surgery measures be implemented by POP surgeons.

## Introduction

Pelvic organ prolapse (POP) refers to the herniation of viscera occupying the pelvis into the vaginal cavity [1]. POP arises from weakness of the anatomic structural supports of the walls of the vagina due to aging, estrogen withdrawal after menopause, birth trauma to the female pelvic floor, stress, and strain from conditions that chronically increase intra-abdominal pressure, such as chronic cough, constipation, and chronic obstructive airway disease, to mention but a few [1,2]. Women with POP may experience alterations in bodily functions: urinary incontinence or obstructed urination in the urinary system, obstructed defecation in the gastrointestinal system, dyspareunia, and penetration difficulty in the reproductive system [2]. Consequently, POP leads to an altered body image, stigma, and social and psychological distress and adversely affects the quality of life of women [3].

The prevalence of POP varies widely, ranging from 3% to 50% depending on whether it was based on self-reports of vaginal bulge sensation symptoms or pelvic examinations [1,2]. Globally, 316 new cases of POP per 100,000 population are detected annually, with low sociodemographic index regions, including sub-Saharan Africa, having higher age-standardized incidences [4,5]. The highest burden of POP in sub-Saharan Africa is among older women, with studies reporting symptomatic POP being more prevalent among women 65 years of age or older [4,5]. The peak prevalence for POP has been reported to be between 70 and 79 years [6].

Moreover, women of advanced age with POP are often frail and suffer underlying medical conditions, like hypertension, diabetes mellitus, ischemic and vulvar heart diseases, lumbosacral spine deformities, and chronic lung diseases [7-9]. These comorbidities make them poor surgical candidates and increase their risk for perioperative complications [10]. A study in Italy reported intraoperative hemorrhage of ≥500 mL in eight out of 65 (12.3%) women aged 80 years or older treated surgically for POP, and a similar study in India reported bladder injury (2.31%), rectal injury (1.85%), and ureteral injury (1.85%) among women 60 years and above operated for POP [8,11]. While studies from high-income settings with technologically advanced health care systems have shown that POP surgery is safe with low rates of perioperative complications in women of advanced age [8,11], there is a paucity of information in low-resource settings. Therefore, in this study, we set out to determine the association between advanced age (≥70 years) and perioperative complications in a low-resource setting.

## Materials and methods

Study design, study population, and setting

We conducted a retrospective review of records among women managed surgically for POP in a total of 30 urogynecological camps conducted over a four-year period between January 2022 and December 2025 across eight regional urogynecological centers in Uganda. The centers included Nakaseke General Hospital, Jinja Regional Referral Hospital, Buyinja Health Centre, Lira Regional Referral Hospital, Mbarara Regional Referral Hospital, Ishaka Adventist Hospital, Bwindi Community Hospital, and Hoima Regional Referral Hospital. The camps were organized and conducted by the urogynecological team of Mbarara University of Science and Technology (MUST) and Mbarara Regional Referral Hospital, with support from the local hospital teams and visiting surgeons.

Inclusion and exclusion criteria

We reviewed a urogynecology care program database of prospectively collected data of women 18 years and above, who underwent surgery for POP during the study period. We excluded women who had missing data for age (primary predictor variable) or perioperative complications (outcome variable).

Data collection and study variables

The urogynecology care program database was reviewed for the period of 2022-2025, and data of women managed surgically for POP during the study period were retrieved using a standardized data collection tool across all the centers. The participant data obtained included the baseline sociodemographic and medical characteristics, type of pelvic floor disorder (PFD), preoperative physical examination findings, intraoperative information, and in-hospital postoperative data.

Categorization and staging of POP was done using the Pelvic Organ Prolapse Quantification (POP-Q) system validated by the International Continence Society (ICS) into stages I, II, III, and IV. Hypertension was based on a diastolic blood pressure of ≥90 mmHg or systolic blood pressure ≥140 mmHg or evidence of prior diagnosis and treatment for elevated blood pressure. Intraoperative complications were clinically assessed by the clinical care team and recorded; they included hemorrhage (intraoperative blood requiring blood transfusion), bladder injury, ureteric injury, and bowel injury. Postoperative complications considered included vaginal cuff hematoma, wound dehiscence, surgical site infection, urine retention (failed voiding that necessitated re-catheterization), re-operation, and prolonged hospitalization (≥7 days postoperatively). Data on postoperative in-hospital complications were also extracted from clinical diagnoses that had been made by the clinical care teams and entered into the standardized postoperative clinical care module of the electronic medical records/database for the urogynecology care program.

Sample size and statistical analysis

Data from all eligible women within the study period were included in this analysis. No prior sample size calculation was done. Data were analyzed using STATA 17 (version 17.0, StataCorp, College Station, TX, USA). Characteristics of participants of advanced age (≥70 years) were compared to those of non-elderly women (<70 years) using a chi-square test. The level of significance was set at 0.05.

The proportion of perioperative complications was compared across the two age groups using a chi-square test with the level of significance set at 0.05. Cramér's V coefficient was used to assess the strength of association. V ≥ 0.5 was considered to indicate a strong effect size.

Bivariable modified Poisson regression analysis with robust standard error was conducted to determine the association between individual predictor variables and the outcome variable. Crude prevalence ratios, 95% confidence intervals, and p-values were reported. Age category and predictor variables with a p-value < 0.2 at bivariable analysis were put into a multivariable modified Poisson regression analysis with robust standard error to test for the independent association between advanced age and perioperative complications. Adjusted prevalence ratios, the corresponding 95% confidence intervals, and p-values were reported. The level of significance was set at 0.05.

## Results

Data from 548 women operated for POP during the study period were available in the database; one was excluded due to a missing record of her age. Therefore, data from 547 women were included in this analysis: 63 (11.5%) with advanced age (≥70 years) and 484 (88.5%) without advanced age (<70 years).

For baseline sociodemographic and medical characteristics, a higher proportion of women of advanced age had hypertension (41.3% vs. 22.9%), did not have formal education (49.2% vs. 28.7%), and did not do work that involved heavy lifting (65.1% vs. 30.5%), compared to their counterparts of the age <70 years. The two groups were not different in terms of other sociodemographic and medical characteristics (Table 1).

**Table 1:** Baseline sociodemographic and medical characteristics HIV: human immunodeficiency virus, x^2^: chi-square test statistic, df, degrees of freedom; *p < 0.05

Variable	Total (n = 547); n (%)	Age ≥ 70 (n = 63); n (%)	Age <70 (n = 484); n (%)	Chi -square test; X^2^ (df)	p-value	Effect size; Cramér's V
Region				20.23 (4)	<0.001*	0.192
Central	43 (7.9)	6 (9.5)	37 (7.6)			
Eastern	38 (7.0)	7 (11.1)	31 (6.4)			
Northern	233 (42.6)	11 (17.5)	222 (45.9)			
Western	228 (41.7)	39 (61.9)	189 (39.1)			
Other (East African country)	5 (0.9)	0 (0.0)	5 (1.0)			
Residence (n = 546)				0.32 (1)	0.571	0.024
Urban	25 (4.6)	2 (3.2)	23 (4.8)			
Rural	521 (95.4)	61 (96.8)	460 (95.2)			
Highest level of education				11.62 (2)	0.003*	0.146
No formal education	170 (31.1)	31 (49.2)	139 (28.7)			
Primary	306 (55.9)	28 (44.4)	278 (57.4)			
Post primary	71 (13.0)	4 (6.4)	67 (13.8)			
Tobacco or cigarette smoking (n = 545)				3.44 (1)	0.064	0.080
No	530 (97.3)	59 (93.7)	471 (97.7)			
Yes	15 (2.8)	4 (6.4)	11 (2.3)			
Alcohol consumption (n = 545)				0.21 (1)	0.647	0.019
No	512 (93.9)	60 (95.2)	452 (93.8)			
Yes	33 (6.1)	3 (4.8)	30 (6.2)			
Work involving lifting heavy masses (n = 539)				41.00 (2)	<0.001*	0.234
No	186 (34.5)	41 (65.1)	145 (30.5)			
Yes	353 (65.5)	22 (34.9)	331 (69.5)			
Body mass index (n = 447)				3.60 (2)	0.165	0.090
Underweight (<18.5)	242 (54.1)	24 (54.6)	218 (54.1)			
Normal weight (18.5–24.9)	92 (20.6)	13 (29.6)	79 (19.6)			
Overweight and obese (≥25)	113 (25.3)	7 (15.9)	106 (26.3)			
History of diabetes mellitus				2.94 (1)	0.086	0.073
No	531 (97.1)	59 (93.7)	472 (97.5)			
Yes	16 (2.9)	4 (6.4)	12 (2.5)			
HIV status (n = 545)				2.59 (1)	0.108	0.069
Negative	487 (89.4)	60 (95.2)	427 (88.6)			
Positive	58 (10.6)	3 (4.8)	55 (11.4)			
Hypertension				9.99 (1)	0.002*	0.135
No	410 (75.0)	37 (58.7)	373 (77.1)			
Yes	137 (25.1)	26 (41.3)	111 (22.9)			

For the baseline POP-related characteristics, a higher proportion of women of advanced age underwent transvaginal hysterectomy, had stage IV POP (46% vs. 22.7%), had a combination of prolapse in the anterior and apical compartments (71.4% vs. 32.7%), and had surgery done in two compartments (73% vs. 38.1%), compared to women less than 70 years of age (Table 2).

**Table 2:** Pelvic organ prolapse-related baseline characteristics POP: pelvic organ prolapse, x^2^: chi-square statistic, df: degrees of freedom, *p < 0.05

Variable	Total (n = 547); n (%)	Age ≥ 70 (n = 63); n (%)	Age <70 (n = 484); n (%)	Chi-square test; X^2^ (df)	p-value	Effect size; Cramér's V
Duration of POP symptoms (n = 353)				1.55 (2)	0.461	0.066
Less than two years	143 (40.5)	21 (47.7)	122 (39.5)			
Two to five years	97 (27.5)	9 (20.5)	88 (28.5)			
More than five years	113 (32.0)	14 (31.8)	99 (32.0)			
POP stage				16.06 (2)	<0.001*	0.171
Stage II	81 (14.8)	6 (9.5)	75 (15.5)			
Stage III	327 (59.8)	28 (44.4)	299 (61.8)			
Stage IV	139 (25.4)	29 (46.0)	110 (22.7)			
Prophylactic antibiotic use (n = 539)				1.21 (1)	0.271	0.047
No	9 (1.7)	0 (0.0)	9 (1.9)			
Yes	530 (98.3)	63 (100.0)	467 (98.1)			
Type of POP surgery				0.66 (1)	0.408	0.035
Repeat surgery	31 (5.7)	5 (7.9)	26 (5.4)			
Primary surgery	516 (94.3)	58 (92.1)	458 (94.6)			
Compartments with POP operated (n = 546)				40.51 (6)	< 0.001*	0.272
Posterior	79 (14.5)	0 (0.0)	79 (16.4)			
Anterior	79 (14.5)	5 (7.9)	74 (15.3)			
Apical	63 (11.5)	6 (9.5)	57 (11.8)			
Anterior and posterior	43 (7.9)	1 (1.6)	42 (8.7)			
Anterior and apical	203 (37.2)	45 (71.4)	158 (32.7)			
Posterior and apical	27 (5.0)	1 (1.6)	26 (5.4)			
All three compartments	52 (9.5)	5 (7.9)	47 (9.7)			
Number of compartments operated (n = 546)				30.98 (2)	<0.001*	0.238
1	201 (36.8)	6 (9.5)	195 (40.4)			
2	230 (42.1)	46 (73.0)	184 (38.1)			
3	115 (21.1)	11 (17.5)	104 (21.5)			
Procedure done for uterine prolapse (n = 312)				7.38 (1)	0.007*	0.154
Hysteropexy	33 (10.6)	0 (0.0)	33 (12.7)			
Transvaginal hysterectomy	279 (89.4)	52 (100.0)	227 (87.3)			
Type of suspension in apical prolapse (n = 335)				1.30 (1)	0.253	0.062
Uterosacral ligament suspension	116 (34.6)	16 (28.1)	100 (36.0)			
Sacrospinous ligament fixation	219 (65.4)	41 (71.9)	178 (64.0)			
Type of anaesthesia				0.01 (1)	0.930	0.004
General anaesthesia	8 (1.5)	1 (1.6)	7 (1.5)			
Spinal anaesthesia	539 (98.5)	62 (98.4)	477 (98.6)			
Duration of surgery				3.75 (2)	0.153	0.083
Less than one hour	95 (17.4)	7 (11.1)	88 (18.2)			
One to two hours	346 (63.3)	39 (61.9)	307 (63.4)			
More than two hours	106 (19.4)	17 (30.0)	89 (18.4)			

The overall prevalence of perioperative complications among women managed surgically for POP during urogynecology camps in Uganda in the study period was 14.26% (n = 78, 95% CI: 11.57-17.46).

The proportion of perioperative complications among women of advanced age was 15.9% (10 /63) and did not statistically differ from the proportion of perioperative complications among women <70 years of age: 14.1% (68 /484); p-value of 0.697 (Table 3).

**Table 3:** Proportions of complications by age category *Chi-square test (degrees of freedom) = 0.15 (1), p-value = 0.697, effect size (Cramér's V) = 0.018. **Due to the very low frequency of individual specific complications, a chi-square test was not done.

Perioperative Complications	Women 70 years or older (n = 63); n (%)	Women younger than 70 years (n = 484); n (%)
Composite of all perioperative complications*	10 (15.9)	68 (14.1)
Specific complications**		
Bladder injury	0	2
Bladder and bowel injury	0	1
Bowel injury	0	4
Vaginal cuff hematoma	0	1
Re-operation	0	1
Long hospital stay (≥7 days)	10	59

In the bivariable analysis, compartment with POP operated upon (cPR 2.76; 95% CI: 1.05-7.23, p = 0.039), and type of anesthesia (cPR 0.37; 95% CI: 0.15-0.93, p = 0.035) were associated with perioperative complications (Tables 4, 5).

**Table 4:** Bivariable analysis for sociodemographic and medical factors BMI: body mass index, DM: diabetes mellitus, HIV: human immunodeficiency virus, Ref: reference category, cPR: crude prevalence ratio, CI: confidence interval, % column percentages

Predictor	Proportion with complications (n = 78), %	cPR (95% CI)	p-value
Age			
Age < 70	68 (87.2)	Ref	
Age ≥ 70	10 (12.8)	1.13 (0.61 – 2.08)	0.695
Education level			
No formal education	23 (29.5)	Ref	
Primary	46 (59.0)	1.11 (0.70-1.77)	0.657
Post-primary	9 (11.5)	0.94 (0.46-1.92)	0.859
Smoking			
No	74 (94.9)	Ref	
Yes	4 (5.1)	1.91 (0.80-4.54)	0.143
Alcohol consumption (n = 545)			
No	70 (89.74)	Ref	
Yes	8 (10.3)	1.77 (0.93-3.37)	0.080
Work involves heavy lifting			
No	29 (37.7)	Ref	
Yes	48 (62.3)	0.87 (0.57-1.34)	0.529
BMI class			
Underweight	13 (20.0)	1.01 (0.56-1.82)	0.985
Normal weight	34 (52.3)	Ref	
Overweight/obese	18 (27.7)	1.13 (0.67-1.92)	0.640
History of DM			
No	76 (97.4)	Ref	
Yes	2 (2.6)	0.87 (0.23-3.25)	0.840
HIV status			
Negative	66 (84.6)	Ref	
Positive	12 (15.4)	1.53 (0.88-2.65)	0.133
Hypertension			
No	54 (69.2)	Ref	
Yes	24 (30.8)	1.33 (0.86-2.07)	0.205

**Table 5:** Bivariable analysis for POP-related factors POP: pelvic organ prolapse, cPR: crude prevalence ratio, CI: confidence interval, % column percentages, *p < 0.05

Predictor	Proportion with complications n = 78, (%)	cPR (95% CI)	p-value
Duration of POP symptoms(n = 353)			
Less than two years	18 (33.3)	Ref	
Two to five years	15 (27.8)	1.23 (0.65-2.32)	0.526
More than five years	21 (38.9)	1.48 (0.82-2.64)	0.188
POP stage			
Stage II	8 (10.3)	Ref	
Stage III	49 (62.8)	1.52 (0.75-3.08)	0.248
Stage IV	21 (26.9)	1.53 (0.71-3.30)	0.278
Type of POP surgery			
Repeat surgery	4 (5.1)	Ref	
Primary surgery	74 (94.9)	1.11 (0.43-2.84)	0.826
Compartments with POP operated (n = 546)			
Posterior	6 (7.7)	Ref	
Anterior	12 (15.4)	2.00 (0.79-5.07)	0.144
Apical	9 (11.5)	1.88 (0.71-5.01)	0.206
Anterior and posterior	9 (11.5)	2.76 (1.05-7.23)	0.039*
Anterior and apical	31 (39.7)	2.01 (0.87-4.64)	0.101
Posterior and apical	3 (3.9)	1.46 (0.39-5.46)	0.571
All three compartments	8 (10.3)	2.03 (0.75-5.51)	0.166
Number of compartments operated			
1	27 (34.6)	Ref	
2	34 (43.6)	1.10 (0.69-1.76)	0.689
3	17 (21.8)	1.10 (0.63-1.93)	0.739
Procedure done for uterine prolapse (n = 312)			
Hysteropexy	4 (8.3)	Ref	
Transvaginal hysterectomy	44 (91.7)	1.30 (0.50-3.40)	0.591
Type of suspension in apical prolapse (n = 335)			
Uterosacral ligament suspension	20 (39.2)	Ref	
Sacrospinous ligament fixation	31 (60.8)	0.82 (0.49-1.38)	0.454
Type of anaesthesia			
General anaesthesia	3 (3.9)	Ref	
Spinal anaesthesia	75 (96.2)	0.37 (0.15-0.93)	0.035*
Duration of surgery			
Less than one hour	14 (18.0)	Ref	
One to two hours	44 (56.4)	0.86 (0.49-1.51)	0.604
More than two hours	20 (25.6)	1.28 (0.69-2.39)	0.438

In the multivariable analysis, advanced age was not significantly associated with perioperative complications (aPR 1.07; 95% CI: 0.57-2.03, p = 0.828), as shown in Table 6.

**Table 6:** Multivariable model for association between advanced age (≥70 years) and perioperative complications POP: pelvic organ prolapse, cPR: crude prevalence ratio, aPR: adjusted prevalence ratio, CI: confidence interval, % column percentages, *p < 0.05

Predictor	Proportion with complications n = 78, (%)	cPR (95% CI)	p-value	aPR (95% CI)	p-value
Age					
Age < 70	68 (87.2)	Ref			
Age ≥ 70	10 (12.8)	1.13 (0.61-2.08)	0.695	1.07 (0.57-2.03)	0.828
Alcohol consumption (n = 545)					
No	70 (89.7)	Ref			
Yes	8 (10.3)	1.77 (0.93 – 3.37)	0.08	1.72 (0.93-3.18)	0.085
POP stage					
Stage II	8 (10.3)	Ref			
Stage III	49 (62.8)	1.52 (0.75-3.08)	0.248	1.44 (0.68-3.04)	0.335
Stage IV	21 (26.9)	1.53 (0.71-3.30)	0.278	1.44 (0.63-3.31)	0.392
Compartments with POP operated (n = 546)					
Posterior	6 (7.7)	Ref			
Anterior	12 (15.4)	2.00 (0.79-5.07)	0.144	2.08 (0.81-5.37)	0.13
Apical	9 (11.5)	1.88 (0.71-5.01)	0.206	1.83 (0.69-4.87)	0.228
Anterior and posterior	9 (11.5)	2.76 (1.05-7.23)	0.039*	2.42 (0.90-6.51)	0.081
Anterior and apical	31 (39.7)	2.01 (0.87-4.64)	0.101	1.82 (0.75-4.40)	0.182
Posterior and apical	3 (3.9)	1.46 (0.39-5.46)	0.571	1.39 (0.35-5.47)	0.638
All three compartments	8 (10.3)	2.03 (0.75-5.51)	0.166	1.91 (0.71-5.15)	0.203
Type of anaesthesia					
General anaesthesia	3 (3.9)	Ref			
Spinal anaesthesia	75 (96.2)	0.37 (0.15-0.93)	0.035*	0.42 (0.17-1.04)	0.064

## Discussion

This study set out to determine the prevalence and association between advanced age and perioperative complications among patients undergoing POP surgery in urogynecology camps in Uganda. The overall prevalence of perioperative complications was 14.26% (n = 78, 95% CI: 11.57-17.46%). Prolonged hospitalization (≥7 days) was the only perioperative complication recorded among women of advanced age. There was no association between advanced age and perioperative complications.

The overall prevalence of perioperative complications in this study was higher than that of 7.1% reported in a retrospective review of 198 POP surgeries conducted in southwestern Uganda [12]. This difference is probably because the current study included prolonged hospitalization (≥7 days) as one of the perioperative complications, which the previous retrospective review did not report on. Studies from developed countries have shown that the cost for POP surgery is enormous, and 71% of that cost is due to hospitalization [13,14]. It is therefore reasonable to report prolonged hospital stay as one of the perioperative complications in our study from a low-income setting. A similar study conducted at Mulago National Referral Hospital, Uganda, reported persistent pain in 35% of women who had undergone POP surgery [9]. However, with regard to intraoperative complications, all reviewed studies in Uganda and Nigeria have consistently shown very low complication rates as found in the current study, with the reported complications being bleeding, iatrogenic bladder, and bowel injury [9,12,15]. One of the studies done in Uganda reported vaginal cuff infection as the most common postoperative complication, observed in 10 (8.3%) of 120 POP surgeries in their cohort [3]. Intraoperative injury to neighbouring pelvic viscera when recognized and repaired primarily is seldom associated with long-term complications [8,16].

There was no significant difference in the proportion of perioperative complications between women of advanced age and women <70 years of age. We did not find directly comparable studies that contrasted perioperative complications in POP surgery between younger and older women. However, from the literature, studies done in women of advanced age generally report as few physical intraoperative and in-hospital POP surgical complications as studies done in the general population [10,16,17].

A multicentre retrospective study in Hong Kong involving 343 women aged 75 years or older who underwent either restorative or obliterative POP surgeries between 2013 and 2018 found that perioperative complications were often minor in their study of women of advanced age and concluded that POP surgery was safe and a viable option in their setting [16]. However, in that study, women undergoing restorative POP surgeries had more complications than those undergoing obliterative procedures (28% vs. 9.3%). The majority of women with medical comorbidities underwent colpocleisis, were operated under spinal anaesthesia, and had shorter operative time (mean of 73.5 minutes) [16]. In comparison, all study participants in our review underwent reconstructive restorative POP surgeries with low rates of complications. The observation of a few perioperative complications in this study was probably because a well-constituted multidisciplinary team of urogynaecologists, residents of obstetrics and gynaecology, anaesthesiologists and residents of anaesthesia and critical care medicine, trained nurses, and a quality improvement team was set up and provided care for these women during urogynaecologic surgical camps. In addition, an obstetrician/gynaecologist or general practitioner (in a few centres) remained in charge of the operated patients at these health facilities until they were discharged, thus ensuring continuity of specialized care. This may have strengthened the postoperative care for these women, thus preventing postoperative complications.

Prolonged hospitalization (≥7 days) was the only complication observed in women of advanced age (≥70 years). A higher proportion of the women with advanced age in our study had hypertension, stage IV POP, transvaginal hysterectomy, or surgery done in two compartments compared to women less than 70 years of age (p-value < 0.05). The higher stage of prolapse, comorbidities, and major prolapse surgeries explain the prolonged hospitalization in this age group. Findings from our study are consistent with a study that analyzed 29,645 same-day discharge female reconstructive pelvic surgeries from the US National Surgical Quality Improvement Program database which found that women who had prolonged length of stay (≥2 days) were more likely to be older (mean age of 59.4 years), were frail (i.e., had more medical comorbidities), or had major surgical procedures or longer operative time [18]. A multicentre randomized controlled trial in China reported a reduction in length of hospital stay in women 60-80 years of age undergoing transvaginal mesh reconstructive surgery for POP when they were offered an enhanced recovery after surgery (ERAS) package to optimize postoperative care [19]. This seems a viable intervention to evaluate and implement in our setting.

Advanced age (≥70 years) was not independently associated with perioperative complications and had an adjusted prevalence ratio of 1.07 (p = 0.828). We did not find directly comparable studies in the literature designed to investigate the association between advanced age and perioperative complications. However, a number of studies have reported physiological and psychological functional decline in elderly women following POP surgery. A secondary analysis of a prospective cohort of 165 women aged 60 years or older undergoing prolapse surgery at the University of Pittsburgh Medical Centre, USA, reported postoperative urinary retention in 47.3% of their study participants at discharge [20]. Structured assessment for postoperative urinary retention following POP surgery by measurement of postvoid residue volume is not routinely practiced in our setting. This complication was not reported in our analysis of a database of POP surgeries in urogynaecologic camps in Uganda. Postoperative urine retention (POUR) may have been missed in our study.

Urinary incontinence has also been reported in women of advanced age following POP surgery. A study in the Netherlands retrospectively followed up 87 women who were 80 years and older and had undergone POP surgery in a period of one to 80 months before the follow-up review: 48 of the 87 women (55.2%) reported urine incontinence [10]. Being retrospective in nature, the study was unable to determine the preoperative continence status of the women in the study. In addition, the study relied on a self-administered postal questionnaire sent with a response rate of 88%. The study could have overestimated the magnitude of urinary incontinence following POP surgery in women of advanced age.

Studies have also reported postoperative delirium and cognitive decline in women of advanced age following POP surgery. A prospective cohort that assessed 72 women aged 60 years or more, two weeks before and after POP surgery, reported postoperative cognitive decline in 33.3% of the study participants [21]. Age in itself did not show a significant statistical association with postoperative cognitive decline in that study. Postoperative cognitive decline is attributed to inhalation anaesthesia, delirium, and decline in organ function following major surgery in frail elderly patients [21,22]. Postoperative delirium has been reported to be as high as 12.1% one week following POP surgery [22].

A prospective cohort study done among 140 Ugandan women to determine the recurrence rate of POP one year after surgery reported a recurrence rate of 25.2%, and women <60 years were twice as likely to have recurrence versus women 60 years and older [17]. Authors of the study reasoned that younger women were more likely to return to strenuous physical activity postoperatively compared to their older counterparts in low-resource settings.

Strength of the study

The study was a multicentre study and therefore gives a fairly accurate representation of perioperative complications encountered in POP surgery in urogynaecologic camps in Uganda and hence can be generalized to similar low-resource settings.

Limitations of the study

The study was a retrospective review of data abstracted from a urogynaecologic surgical database and only considered intra-operative and in-hospital postoperative complications. Surgical complications that are not routinely assessed for and those that occur remotely could have been missed. Hence, the magnitude of complications could have been under-estimated. The study had missing data for some of the variables, and in addition, it did not have an a priori calculation of sample size. However, the study analyzed and reported on data of all women operated for POP during the study period of four years. The authors recognize that some important clinical data in surgical care for elderly women, such as frailty assessment, comorbidity severity assessment, physical function assessment, and American Association of Anesthesiologists (ASA) pre-anesthetic grading, were not available in this retrospective analysis. The study, therefore, may have provided an incomplete clinical description of the elderly women operated for POP in urogynecology surgical camps.

## Conclusions

In our analysis of POP surgeries done in urogynecological surgical camps in Uganda, perioperative complications were infrequent, prolonged hospitalization was the only perioperative complication identified among elderly women, and advanced age was not associated with perioperative complications. We recommend that surgeons offer restorative surgical management as a viable, safe option to treat POP in women of advanced age in surgical camp settings. ERAS measures should be instituted in POP surgical care to prevent prolonged hospitalization.
